# Awareness, knowledge and treatment decisions for erosive tooth wear: A case‐based questionnaire among Danish dentists

**DOI:** 10.1002/cre2.339

**Published:** 2020-10-30

**Authors:** Diana Mortensen, Aida Mulic, Ulla Pallesen, Svante Twetman

**Affiliations:** ^1^ Department of Odontology, Faculty of Health and Medical Sciences University of Copenhagen Copenhagen Denmark; ^2^ Institute of Dental Materials (NIOM) Oslo Norway

## Abstract

**Objective:**

To examine the knowledge and experience of erosive tooth wear (ETW) among Danish dental practitioners and, based on two cases, explore their treatment decisions.

**Methods:**

We sent a validated questionnaire electronically to all active members of The Danish Dental Association and The Association of Public Health Dentists in Denmark. The questionnaire had two parts; the first focused on scoring, recordkeeping, knowledge and experience of ETW. The second part presented two patients with different severity of erosive lesions to explore the dentists preventive and restorative treatment decisions.

**Results:**

We received 442 answers from 4,490 potentially eligible dentists in Denmark (response rate 9.8%). The majority (78%) was female and the median age was 44 years. Nearly all respondents (97%) registered ETW in the charts and 49% recorded “always” or “often” the patients' diet history, most commonly with aid of interviews. The respondents perceived the prevalence of ETW to be higher today than 10–15 years ago and male patients (15–25 years) appeared more affected than females. The majority (82%) thought that they usually found the probable cause of the condition with carbonated beverages being the most common factor. The treatment included dietary guidance, soft tooth brushing with non‐abrasive fluoride toothpaste, topical fluoride applications and direct composite restorations.

**Conclusion:**

The majority of Danish dentists taking part of this survey had adopted a minimally invasive approach for the management of erosive tooth wear in young adults. There was however room for improvements in diagnosis, scoring and case documentation.

## 1 INTRODUCTION

Dental erosion is the chemical loss of mineralized tooth substance caused by the exposure to acids not derived from oral bacteria (Schlueter et al., 2020). The acids are either of extrinsic or intrinsic origin but since dental erosion commonly occurs in conjunction with abrasive processes, the term “erosive tooth wear” (ETW) is commonly used (Lussi & Carvalho, 2014). Based on a systematic review, the average prevalence of ETW in permanent teeth of children and adolescents was 30.4%, but the occurrence was highly influenced by methodological and diagnostic factors (Salas, Nascimento, Huysmans, & Demarco, 2015). The mild and the moderate stages of ETW are most often addressed through secondary prevention involving risk assessment, personalized counseling and non‐operative interventions (Loomans et al., 2017). The advanced stages with functional and esthetic compromises may require restorative treatment in addition to the preventive approach (Carvalho et al., 2015).

Although the understanding of ETW and its contributing factors has increased over the recent decades, the diagnosis, classification and management seem to be a challenge in everyday practice (O'Toole et al., 2018). In order to improve education and clinical decision‐making, it is important to understand the awareness, experience and preferred treatment strategies among dental health professionals in everyday practice. Previous studies from Norway and Iceland have indicated that dentists generally are well educated with respect to diagnosis and treatment of ETW although dietary and salivary analyses had low priority. (Mulic, Árnadòttir, Jensdottir, & Kopperud, 2018; Mulic, Vidnes‐Kopperud, Skaare, Tveit, & Young, 2012). A recent questionnaire among dentists from Germany displayed that the decision‐making was mainly dependent on the lesion depth, size of affected surfaces and the presence of pain (Kanzow, Biermann, & Wiegand, 2019). However, there is a gap of knowledge concerning the awareness and preferred treatment strategies among practicing dentists in Denmark and a national survey would be a first step in the quality assurance process. The aim of this study was therefore to gain information about Danish dental practitioners' experience and knowledge of ETW with aid of an electronic questionnaire. Two illustrated adult cases with different severity of the condition were included in the survey to explore the respondents' preferences and treatment decisions.

## 2 STUDY POPULATION AND METHODOLOGY

In August 2018, a link to the questionnaire designed with aid of the software tool SurveyXact (Rambøll, Aarhus, Denmark) was sent electronically to dentists in Denmark. The survey was sent by e‐mail to all members of the Association of Public Health Dentists in Denmark (ATO), and with a regular newsletter to the members of the Danish Dental Association (TF), covering dentists working in the private sector. In addition, we uploaded the questionnaire on a closed Facebook group consisting of practicing dentists in Denmark. The link to the questionnaire was distributed twice to the members of ATO and TF, followed by a reminder after 2 weeks, and the questionnaire was posted two times at the Facebook group. In August 2018, there were 4,490 registered dentists in Denmark but due to The General Data Protection Regulation (GDPR) in the European Union, we were not allowed to contact them directly via e‐mail.

### 2.1 The questionnaire

The Danish questionnaire used in this study was a modified version of questionnaire used in Norway in 2011 (Mulic et al., 2012), but almost identical to the one used in Iceland (Mulic et al., 2018). The questionnaire collected information on the respondents' sex, age, home region, and type of dental practice, and to which extent the respondents were involved in the diagnosis and treatment of ETW. We pooled all dentists aged 65 years and above into one group after consultation with the Danish Data Protection Agency.

**FIGURE 1:**
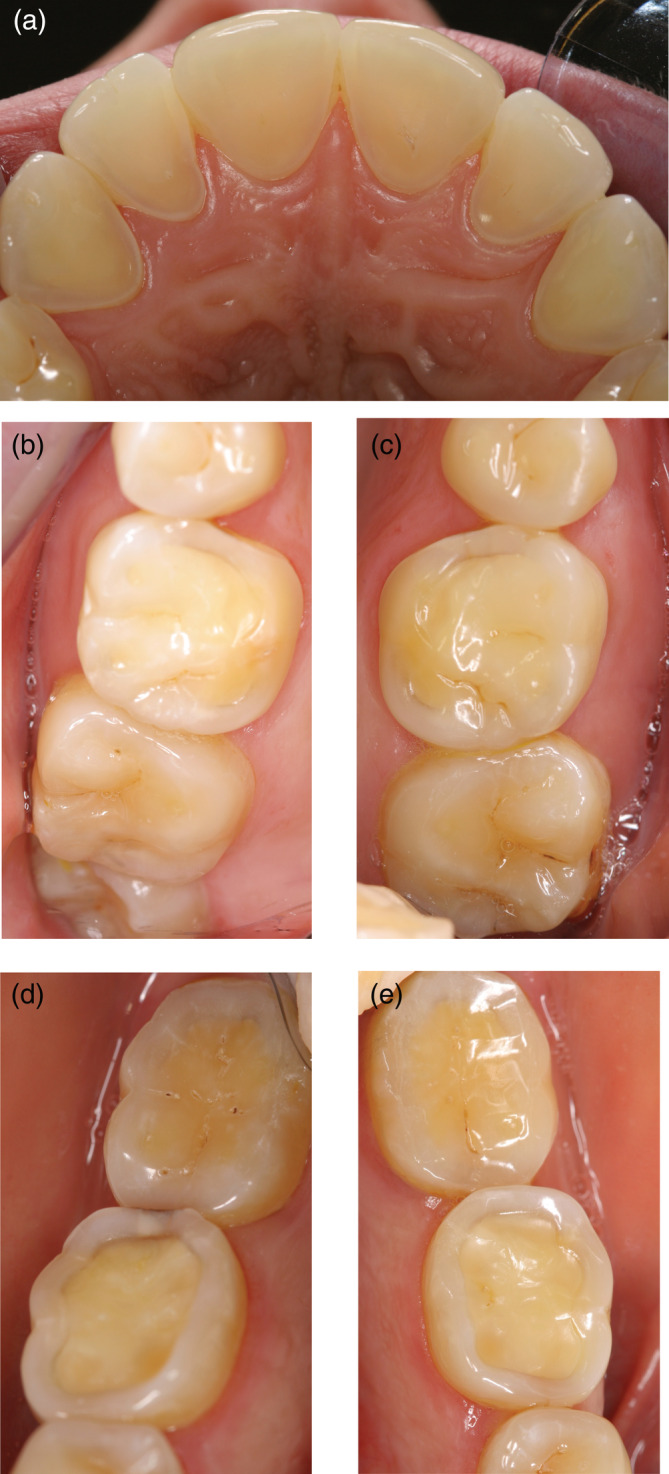
(a–e) Patient case 1. A 28‐year‐old woman who had an eating disorder with vomiting as a teenager, but she is now healthy. (a) Palatal surfaces of the upper incisors, (b) occlusal surfaces of the upper right first and second molars, (c) occlusal surfaces of the upper left first and second molars, (d) occlusal surfaces of the lower right first and second molars and (e) occlusal surfaces of the lower left first and second molars

**FIGURE 2:**
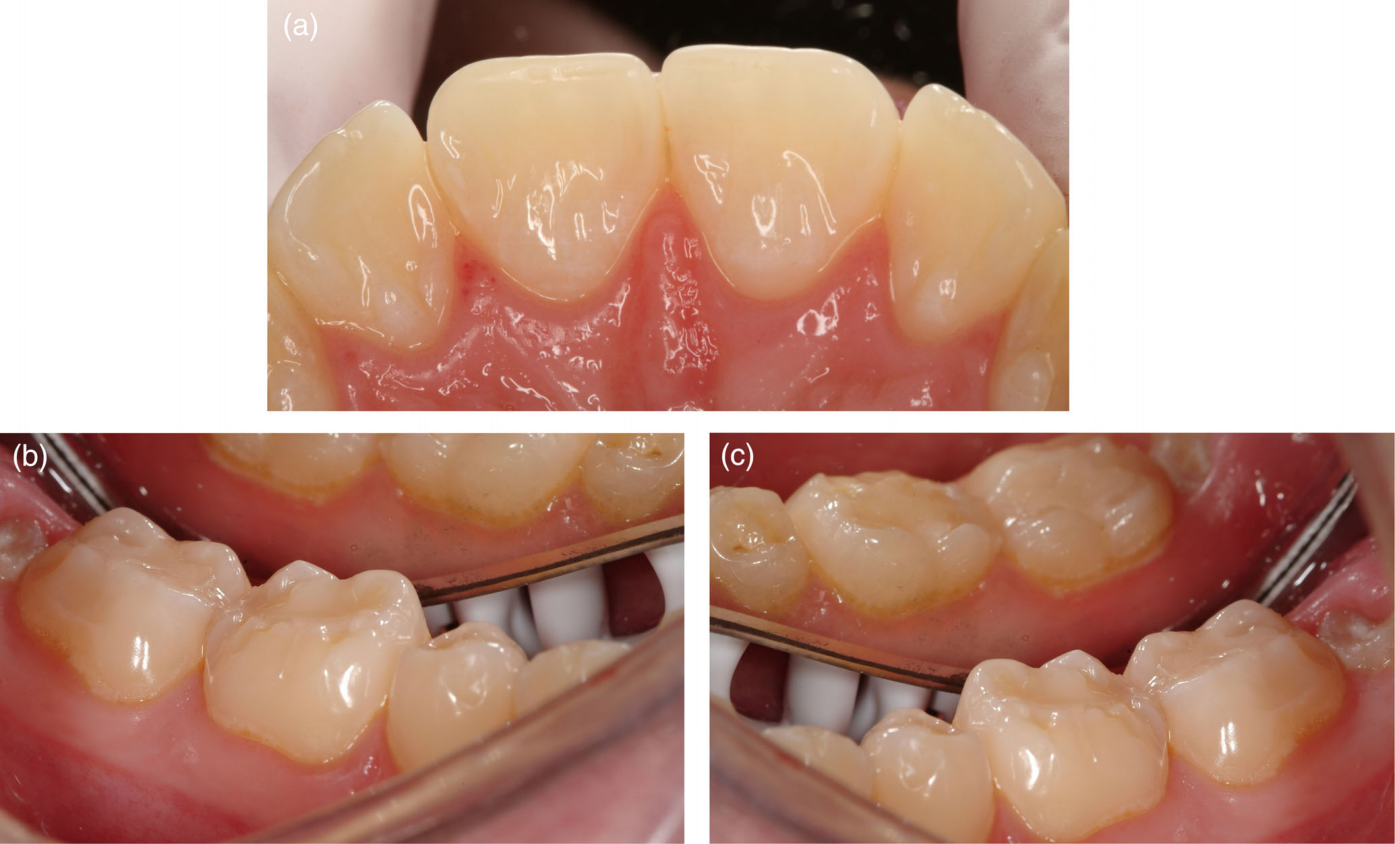
(a‐c) Patient case 2. A 25‐year‐old man who suffers from hypersensitivity in the lower molar region. He consumes large amounts of carbonated beverages. (a) Palatal surfaces of the upper incisors, (b) occlusal surfaces of the lower right first and second molars and (c) occlusal surfaces of the lower left first and second molars

The questionnaire included two parts; Part 1 focused on how dentists score ETW among their patients aged 15–26 years and how this assessment is documented in the dental records. This first part also captured the dentists' knowledge about location, prevalence and probable cause of the erosive lesions. Part 2 presented two adult patient cases (Figures 1 and 2) and the respondents were asked to note the preferred preventive care and treatment decisions. To submit the questionnaire, all mandatory questions had to be completed.

### 2.2 Statistical analyses

We processed the data with aid of the IBM‐SPSS software (version 24.0; Chicago, IL, USA) and presented the results with descriptive statistics.

### 2.3 Ethical considerations

The questionnaire was approved by the Danish Data Protection Agency and the project was exempted from ethical application after contact with the Regional Ethical committee in Copenhagen. The participation was voluntary and SurveyXact ensured the anonymity.

## 3 RESULTS

We received answers from 442 dentists, but 23 respondents declared that they did not work with ETW. Thus, 419 dentists were included in the final analyses, giving a response rate of 9.8% in relation to number of potentially eligible dentists in Denmark. The majority (78%) were women. The age ranged between 23 years and >65 years; the median age was 44 years.

### 3.1 Scoring and documentation of erosive lesions

Almost all respondents (97%) reported that they registered ETW in the patient's charts; 37% used a simple two‐graded system scoring erosions within the enamel or the dentin, while 27% used a more detailed system (e.g., Basic Erosive Wear Examination). The severity of the lesions was described in words by 45%. Among those using a structured system, the erosions were scored on surface level by 22% and on tooth‐ or individual level by 14% and 10%, respectively. Seventeen percent stated that they “often” took clinical photos to document the condition, but very few took study models or performed any saliva tests (Table 1). Fifty‐seven percent considered the salivary flowrate as normal, 9% less than normal, while “do not know” accounted for 34%. Almost half of the dentists (49%) answered that they “always” or “often” recorded the diet history in patients with ETW (Table 1). In contrast, 25% stated that they never recorded the diet of their ETW patients. The method for collecting diet information varied from a pre‐coded chart (9%) to a system where the patients were asked to note every food intake (amount, type, time) during a pre‐defined timeframe (29%). However, most dentists (62%) claimed that they used different interviewing techniques and wrote an explanatory text in the dental record.

**TABLE 1:** Use of clinical photographs, study models and salivary tests to document the stage and background factors of ETW

Measure	“Always”	“Often”	“Sometimes”	“Never”
Clinical photos	—	16.7	59.6	23.6
Study models	—	4.7	51.2	44.1
Saliva flow measured	—	0.7	3.1	96.2
Diet history	28.6	19.6	26.7	25.1

*Note:* Values denote percent (*n* = 419).

### 3.2 Prevalence, location and causes of erosive lesions

A majority (66%) of the experienced dentists reported that they perceived a higher prevalence of ETW 2018 than 10–15 years ago, while 23% were not sure. The most common locations were the palatal surfaces of the upper anterior teeth (71%) followed by the occlusal surfaces of the first mandibular molars (65%) and buccal surfaces of the upper anterior teeth (31%). Gender differences in prevalence were reported by 49% of the dentists; the majority thought that male patients (15–25 years) were more affected than females but 35% saw no gender differences. The majority of the dentists (82%) thought that they usually found the probable cause of the erosive lesions, 17% occasionally and only 2% answered that they seldom found a probable cause or did not know. The most common cause was carbonated beverages (98%) followed by acidic juices (46%), sport drinks (46%) and fruit (28%). The dentists also frequently reported reflux (24%) and eating disorders (13%) among the causative factors.

The question concerning a possible co‐morbidity of erosive lesions and dental caries divided the respondents; half of them (49%) thought that this was true, while 45% did not think that patients with ETW had a higher prevalence of caries. Most of the respondents chose to manage the patients with ETW in their own clinic (78%), 10% referred them to another dentist, specialist or university clinic. The remaining 12% referred only the most advances cases.

### 3.3 Treatment decisions

The two patient cases represented a different severity of ETW. The distribution of the general advice and the preferred treatment of choice are shown in Tables 2, 3 and 4. Information concerning non‐erosive eating and drinking habits was almost mandatory given together with oral hygiene reinforcement with a soft brush and a non‐abrasive fluoride toothpaste. Topical fluoride applications were the preferred non‐operative option while direct composite restorations were the dominating restorative treatment, in particular for the first molars. Less than 3% of the respondents considered ceramic laminates/onlays or crown restorations. There were no principal differences in the treatment decisions between the case with more severe erosions and the case with less severe erosions.

**TABLE 2:** *What type of advice would you give this patient?* More than one choice was possible and the values in the table denote the frequency of dentists' advice expressed as per cent (*n* = 419)

Advice	Patient case 1		Patient case 2	
	*n*	%	*n*	%
Information about good dietary and drinking habits	347	82.8	419	100
Information about good brushing techniques and habits	222	53	263	62.8
Recommend brushing with fluoride	313	74.7	296	70.6
Recommend specific toothpaste or rinse	50	11.9	95	22.7
Refer to specialist, faculty clinic or other dentist	34	8.1	7	1.7
Recommend rinsing with chlorhexidine	2	0.5	0	0

**TABLE 3:** *How would you treat the teeth in the maxillary anterior region and the posterior teeth?* More than one choice was possible and the values in the table denote the frequency of dentists' choice of treatment expressed as per cent (n = 419)

Treatment decision	Patient case 1					
	Incisor		First molar		2nd molar	
	Central	Lateral	Lower	Upper	Lower	Upper
No treatment	41.3	32.9	17.7	23.9	32.7	44.4
Topical fluoride application^a^	21.7	20.5	16.5	17.2	20.0	21.0
Bonding material	5.5	7.2	7.6	8.4	6.4	5.0
Flow	5.7	7.6	8.4	8.1	7.2	5.0
Glass ionomer	0.2	0.0	0.7	0.5	0.2	0.2
Compomer	1.2	1.0	2.1	2.1	1.7	1.0
Composite	30.5	40.6	56.1	48.2	37.0	25.5
Restore with ceramic laminate/facet/inlay/onlay	0.7	1.2	1.2	0.5	1.4	0.7
Restore with crown	0.0	0.0	2.6	1.0	1.7	0.7

^a^ For example, 2% sodium fluoride solution, 5% sodium fluoride varnish, sodium fluoride (APF) gel.

**TABLE 4:** *How would you treat the teeth in the maxillary anterior region and the mandibular posterior teeth?* More than one choice was possible and the values in the table denote the frequency of dentists' choice of treatment, expressed as per cent (*n* = 419)

Treatment decision	Patient case 2			
	Incisor		First molar	2nd molar
	Central	Lateral	Lower	Lower
No treatment	20.8	32.2	12.9	19.6
Topical fluoride application^a^	32.2	36.3	36.0	40.0
Bonding material	10.7	8.1	11.7	10.2
Flow	11.5	7.4	11.9	10.3
Glass ionomer	0.0	0.0	0.5	0.7
Compomer	1.0	0.2	2.4	1.9
Composite	49.4	33.2	53.5	41.1
Restore with ceramic laminate/facet/inlay/onlay	2.1	1.9	1.0	1.4
Restore with crown	0.0	0.2	1.0	1.2

^a^ For example, 2% sodium fluoride solution, 5% sodium fluoride varnish, fluoride (APF) gel.

## 4 DISCUSSION

Dental erosions are common among children and adolescents but the condition has gained comparatively little attention from the research community. In fact, a comprehensive mapping of systematic reviews classified diagnosis, prevention and treatment of dental erosions and tooth wear as knowledge gaps (Mejàre et al., 2015). It was therefore of interest to investigate the Danish dentists' knowledge, opinions/beliefs and clinical decision‐making concerning ETW. The strength of the study was that a previously validated electronic questionnaire was used that allowed direct comparisons with findings from other countries (Mulic et al., 2012; Mulic et al., 2018). The major weakness was obviously the limited response rate (~10%), which may have introduced both selection and response bias. There is a possibility that the most interested and recently educated dentists were more prone answer the survey. Therefore, our findings may not be representative for the 90% non‐responding dentists who may have no idea about the condition. The present response rate was lower than in the corresponding investigations conducted in Norway and Iceland (Mulic et al., 2012; Mulic et al., 2018), but clearly higher than in a recent report from Germany (Kanzow et al., 2019). It must however be strongly underlined that our calculated response rate was the theoretical “worst case scenario”. The figure could be higher in reality as we do not know how many dentists that actually took notice of the invitation. Due to the current GDPR within EU, the link to the questionnaire was included in an e‐mail or a regular newsletter via the domestic dental associations and not directly sent to the dentists. For the same reason, reminders could not be personalized or targeted to non‐responders. Another technical shortcoming was that the survey required a personal computer rather than a smart phone or tablet. Taken together, we think that the legal and technical issues illustrate some of the obstacles that are associated with this kind of internet‐based research.

The overrepresentation of female dentists might also have affected the results. Previous research have indicated that female dentists tend to emphasize more on preventive oral health behavior among their regular check‐up patients than male dentists (Takeuchi, Noguchi, Nakai, Ojima, & Yamashita, 2020). Furthermore, female dentists seem to choose preventive therapies for initial dental caries while male dentists more often prefer to treat enamel caries invasively (Kakudate et al., 2012; Riley et al., 2011). It is possible that a similar gender difference is relevant for ETW; we found a tendency that male respondents suggested crowns and veneers on the upper anterior teeth in case 2 more often than females (6% vs. 1%). This may give a hint that gender may have a significant impact on treatment options also in the field of ETW.

The general impression of this survey indicated that the respondents in general have adopted a restrictive “minimal” treatment philosophy based on fluorides and tissue‐preserving techniques. At the same time, there seemed to be notable room for improvements. Concerning the assessment, most dentists were confident that they were able to identify the cause, or causes, of the condition with overconsumption of carbonated drinks as the main background factor. This was interesting since only half of the respondents actually stated that they took a diet history “often” or “always.” In addition, very few used a pre‐coded chart for collecting dietary habits and saliva tests were utilized by very few. The low priority of collecting data on eating and drinking habits was consistent with previous reports from Norway and Iceland (Mulic et al., 2012; Mulic et al., 2018). The reason for this can be that dentists find 3‐day diet histories and saliva samplings too time‐consuming to evaluate and/or handle.

We found that virtually all respondents recorded ETW in the dental records, and 60% used any form of objective scoring system. This was encouraging, albeit only 34% of them did it on tooth or surface level. Effective management of ETW includes screening for early signs of the condition and evaluating all etiological factors, including the medical conditions (Carvalho et al., 2015). Thus, a comprehensive patient history, detailed scoring and registration of the erosive lesions are key elements for the care‐planning, preventive treatment and monitoring of the condition. In this context, the relatively low frequency of dentists that captured clinical photos or utilized cast study models was a concern. It is possible that time‐constraints may affect such scoring and documentations but, unfortunately, the questionnaire did not allow such clarifications. The dentist's opinion that ETW was more frequent among males than females was in accordance with the international literature (Mulic et al., 2016; Skalsky Jarkander, Grindefjord, & Carlstedt, 2018; Van't Spijker et al., 2009). A majority (66%) of the dentists assumed that ETW was more prevalent today than 10–15 years ago, but this may be due to an increased awareness of the condition. It is not clear whether or not the prevalence is changing; a study from Norway indicated that the prevalence of ETW was unchanged over 30 years (Stenhagen, Berntsen, Ødegaard, Mulic, & Tveit, 2017), while others reports have shown an increasing presence of erosions over the past decades (Jaeggi & Lussi, 2014; Tschammler, Müller‐Pflanz, Attin, Müller, & Wiegand, 2016). However, due to different scoring systems, samples and examiners, it is difficult to compare the prevalence of ETW from different studies (Carvalho et al., 2015; Jaeggi & Lussi, 2014).

A common comment from the respondents was that that not enough case information was available for appropriate treatment decisions and that the photos did not allow proper assessments of the occlusion or parafunctions. Nevertheless, the interventions and treatment suggestions were relatively uniform. For the secondary prevention, good dietary and drinking habits and advocating a gentle tooth brushing technique with fluoridated non‐abrasive toothpaste was the preferred options. Reducing the frequency of erosive foods and beverages seemed rational since almost all respondents blamed the condition on carbonated soft drinks. Addressing the patients diet habits and educate patients on potential acidic interactions, such as medically induced acidic conditions, is in harmony with the current consensus recommendations (Passos, Melo, Park, & Strassler, 2019), while the evidence for linking the tooth brushing habits with ETW is more uncertain (Carvalho et al., 2015). However, 12% recommended also specific products, such as toothpaste or mouth rinse containing stannous fluoride, which may slow the progression of ETW (Stenhagen, Hove, Holme, & Tveit, 2013).

Even though there is no standard treatment for damages caused by ETW, the management should be based on the individual need with emphasis on the minimal invasive intervention (Carvalho et al., 2015). For the two cases, it was obvious that the majority of the responding dentists favored the least invasive therapy. Composites were the overall preferred material and the first permanent molars were the most frequently restored. Interestingly, the alternative to restore teeth with aid of ceramic laminate veneers, indirect onlays or crowns was close to zero for all teeth except for the first lower molar in the first case, emphasizing the minimal intervention approach among the respondents. As a rule, one should use the least invasive therapy; direct procedures are less invasive than indirect and therefore composites have been recommended (Carvalho et al., 2015). One additional explanation for the low restorative rate could be that claims of hypersensitivity and pain are hard to “visualize” in a questionnaire. The option to treat ETW with topical fluoride varnish applications or solutions, was frequently reported by respondents, which was somewhat surprising in the light of current evidence. According to systematic reviews, the effect of fluoride toothpaste, fluoride mouth rinses and fluoride varnishes in preventing and treating dental erosion is inconclusive (Abdelwahed, Temirek, & Hassan, 2019; Mohammed & Dusara, 2013; Zini, Krivoroutski, & Vered, 2014). It is possible that the remineralizing properties documented from the reversal of early caries lesions have spilled over to the management of erosions but most importantly, a beneficial effect of fluorides cannot be excluded (Hove, Stenhagen, Mulic, Holme, & Tveit, 2015).

## 5 CONCLUSION

Within the limits of the present survey, we conclude that the responding Danish dentists seem to have adopted a minimally invasive approach for the management of ETW in young adults; the preventive focus was soft drink reduction while the first restorative option was direct composites. There was however room for improvements concerning the diagnosis, clinical scoring and case documentation.

## AUTHOR CONTRIBUTIONS

The study conception came from A.M. and U.P. but all authors were involved in the final design of the project. D.M. carried out the practical work and performed the analyses and interpretation of the data together with S.T. All authors participated in drafting and writing the manuscript, as well as read and approved the final manuscript.

## CONFLICTS OF INTEREST

The authors declare no potential conflict of interest. The authors' institutions funded the study.

## Data Availability

The data that support the findings of this study are available from the corresponding author upon reasonable request.
